# Effect of *Bifidobacterium animalis* subsp. *lactis* GCL2505 on the physiological function of intestine in a rat model

**DOI:** 10.1002/fsn3.344

**Published:** 2016-02-08

**Authors:** Ryo Aoki, Sayaka Tsuchida, Yuri Arai, Konatsu Ohno, Tomohiko Nishijima, Takashi Mawatari, Yumiko Mikami, Kazunari Ushida

**Affiliations:** ^1^ Instutute of Health SciencesEzaki Glico Co. Ltd.OsakaUtajimaJapan; ^2^ Glico Dairy Products Co. Ltd.AkishimaTokyoJapan; ^3^ Graduate School of Life and Environmental ScienceKyoto Prefectural UniversityShimogamoKyoto606‐8522Japan

**Keywords:** *Bifidobacterium animalis* ssp. *lactis*, bile acids, constipation, IgA, mucin, Probiotics, proliferation

## Abstract

*Bifidobacterium animalis* ssp. *lactis* GCL2505 has been shown to proliferate in the human intestine. The intestinal dynamics and physiological effects of GCL2505 as well as the mechanism underlying proliferation in the gut were investigated. GCL2505 showed markedly higher resistance to free bile acids (cholic and deoxycholic acids) than other bifidobacterial species. The intestinal dynamics of GCL2505 and *B. longum* ssp. *longum* JCM1217^T^ was compared. The level of *B. animalis* ssp. *lactis* in the GCL2505‐administered group was remarkably higher than that of *B. longum* in the JCM1217^T^‐administered group. The distribution of *B. animalis* ssp. *lactis* through the intestine of the GCL2505‐administered group revealed that GCL2505 proliferated in the cecum. The physiological effects of GCL2505 and JCM 1217^T^ were investigated. The cecal IgA level in the GCL2505‐administered group was significantly higher than that in the nontreated control group. In contrast, the JCM 1217^T^‐administered group did not manifest any change in the cecal IgA level. Mucin excretion in the GCL2505‐administered group was significantly higher than that in the JCM 1217^T^‐administered group. The thickness of the sulfomucin layer of the colon in the GCL2505‐administered group tended to be higher than that in the JCM 1217^T^‐administered group. In a loperamide‐induced constipation model, fecal excretion in the GCL2505‐administered group was significantly increased compared with that in the loperamide‐treated control group. Short‐chain fatty acid concentration in the GCL2505‐administered group was significantly higher than that in the loperamide‐treated control group. These results indicate that the level of proliferation of probiotics in the intestine correlates with the magnitude of host physiological responses, such as IgA production and mucin secretion, which possibly affect gastrointestinal functions such as bowel movement to counteract constipation. GCL2505 exhibits high tolerance to secondary bile acids, which partially explains its higher rate of proliferation in the large intestine.

## Introduction

The human intestinal tract harbors a large, active, and complex community of microorganisms comprising over 500 different taxons (Selkirov et al. 2010). Gut microbiota play several significant health beneficial roles for their host such as the digestion of food, metabolism of endogenous and exogenous compounds, immunomodulation, and protection from pathogenic infection (Clemente et al. 2012; Nicholson et al. 2012). They continuously produce large amounts of various bioactive compounds including short‐chain fatty acids (SCFAs), which have been considered to stimulate colonic cell proliferation, mucins, and IgA (Ichikawa and Sakata 1998; Shimotoyodome et al. 2000a; Willemsen et al. 2003; Morita et al. 2004). Accordingly, the deterioration of gut microbiota often induces not only various acute disorders such as diarrhea (Young and Schmidt 2004; Khoruts et al. 2010) but also chronic disorders such as obesity (Turnbaugh et al. 2006, 2009; Vijay‐Kumar et al. 2010). According to these findings, maintaining normal or healthy gut microbiota is now recognized as a key issue in promoting general health.

Probiotics are defined as “live microorganisms that, when administered in adequate amounts, confer a health benefit on the host” (FAO/WHO, 2002), of which *Bifidobacterium* and *Lactobacillus* are representative examples. The importance of probiotics on the issues of promoting general health is now well recognized due to their positive effects on intestinal microbial imbalance, suppression of pathogens, prevention and treatment of intestinal and other disorders, inflammatory bowel disease, diarrhea, infection, colon cancer, constipation, atopic diseases, and obesity (Isolauri et al. 2001; Guarner and Malagelada 2003; Nomoto 2005; Shioiri et al. 2006; De Preter et al. 2007; Miyazaki and Matsuzaki 2008; Yonejima et al. 2013). In particular, numerous attempts have been made to improve intestinal disorders such as constipation and diarrhea by probiotics (Yaeshima et al. 1997; Matsumoto et al. 2000; Shimakawa et al. 2003; Larsen et al. 2006). Many such effects are based on the metabolic activities of probiotic strains. This indicates that probiotics are required to survive even in low pH environments in the stomach and the antimicrobial activity of bile salts in the small intestine. Probiotic strains have been selected according to survivability against gastric and duodenal bile acids (Fuller 1997). Primary bile acids are deconjugated in the small intestine and transformed into secondary bile acids by colonic microbiota (Ridlon et al. 2006). Secondary bile acids such as deoxycholate and lithocholate are highly toxic to intestinal microorganisms (Kurdi et al. 2006) and are thought to play important roles in the modulation of gut microbiota and host homeostasis (Islam et al. 2011; Yokota et al. 2012; Yoshimoto et al. 2013). However, the effects of secondary bile acids on the growth or survival of probiotics are largely unknown.

*Bifidobacterium animalis* subsp. *lactis* GCL2505 originated from healthy human intestines and is used in fermented milk products in the Japanese market. We previously showed that GCL2505 reaches the intestine in a viable form and subsequently proliferates to increase the total number of intestinal bifidobacteria (Ishizuka et al. 2012). However, the precise location where the GCL2505 grew during the passage of the intestine is unknown. Accordingly, mechanisms derived from host–GCL2505 interaction underlying the actual proliferation of GCL2505 in the gut remain unclear.

In this study, we investigated the characteristics of *B. animalis* ssp. *lactis* GCL2505 in vitro and in vivo compared with those of other bifidobacteria such as *B. longum* JCM 1217^T^, a type strain of a bifidobacterial species, which has been widely used as a probiotic.

## Methods

### Bacterial strains

*B. animalis* ssp. *lactis* GC2505 was obtained from Glico Dairy Products Co., Ltd. (Tokyo, Japan). *B. longum* ssp. *longum* JCM 1217^T^, *B. adolescentis* JCM 1275^T^, *B. catenulatum* JCM 1194^T^, *B. longum* ssp. *infantis* JCM 1222^T^, and *B. breve* JCM 1192^T^ were obtained from the Japan Collection of Microorganisms (RIKEN, Tsukuba, Japan). For animal experiments, bifidobacteria, anaerobically cultured on GAM broth (Nissui, Tokyo, Japan) at 37°C for 24 h, were washed and suspended in sterile saline.

### Animals

The animal experiments were conducted in accordance with the guidelines for studies with laboratory animals of the Kyoto Prefectural University Experimental Animal Committee or Institutional Animal Care and Use Committee of Ezaki Glico Co., Ltd. Male Fischer (*N *=* *344) rats were purchased from Japan SLC (Hamamatsu, Japan) at 7 weeks old. They were individually housed in steel wire cages under a controlled temperature (25**°**C) and a 12‐h light:12‐h dark cycle.

#### Animal experiment 1

After acclimatization, the rats were fed AIN‐93M (25 g/day). The rats were divided into four groups (*N *=* *4–6 each): (1) transient‐GCL2505 group, (2) transient‐JCM 1217^T^ group, (3) consecutive‐GCL2505, and (4) consecutive‐JCM 1217^T^ group (Fig. S1A). In the transient feeding group, GCL2505 or JCM 1217^T^ was administered (10^10^ cfu/day, suspended in 0.5‐mL sterile saline) with an oral feeding needle for 2 days followed by sterile saline for 6 days to wash out the ingested bifidobacteria. The rats in the consecutive ingestion groups were administered GCL2505 or JCM 1217^T^ for 8 consecutive days. All excreted feces were collected from the first day of the ingestion of bifidobacteria, every 3 h, to the eighth day of the experiment. On the eighth day, the intestinal contents were collected from each individual. Bacterial DNA from 25 mg of fecal sample (or standard culture) was extracted using Quick Gene DNA tissue kit S (Kurabo, Tokyo, Japan) according to the manufacturer's protocol.

The number of administered bifidobacteria in the feces and intestinal contents was analyzed by quantitative real‐time polymerase chain reaction (PCR) using *B. animalis* ssp. *lactis* or *B. longum* ssp*. longum* species‐specific primers (Malinen et al. 2003; Matsuki et al. 2004). PCR amplification and detection were performed with the Light Cycler 480^®^ (Roche Applied Science Indianapolis, IN). Each reaction mixture (10 *μ*L) comprised 5 *μ*L of SYBR premix Ex *Taq* (Takara Bio, Otsu, Japan), 0.2 *μ*L of each primer (10 pmol/L), 1 *μ*L of DNA template, and distilled water. The amplification program for primers comprised one cycle of 95°C for 30 sec, then 40 cycles of 95°C for 30 sec followed by 63, 60, or 55°C for 30 sec, and ending with 72°C for 50 sec. DNA was extracted from 10‐fold serial dilutions of the cultures and was used as a standard for the quantification of bifidobacterial species in the samples.

#### Animal experiment 2

After acclimatization, 15 rats were divided into three groups (*N *=* *5 each): control group, GCL2505 group, and JCM 1217^T^ group. The rats in the GCL2505 and JCM 1217^T^ groups were orally administered bifidocterium (10^10^ cfu/day, suspended in 0.5‐mL sterile saline) daily for 7 days (Fig. S1B). Feces excreted and kept for 24 h were collected on the seventh day of the experiment. After suspension in saline at 4°C for 24 h, the fecal samples were homogenized and centrifuged at 10,000*g* for 10 min to obtain the supernatant. O‐linked oligosaccharide chains were measured using a fluorometric assay to estimate fecal mucin as described elsewhere (Crowther and Wetmore 1987). Standard solutions of mucin from the bovine submaxillary gland (Sigma Chemicals, St. Louis, MO) were used to calculate the amount of fecal mucin. On the eighth day of the experiment, cecal contents and whole colonic tissues were collected from each rat. The colon tissues and contents were immediately frozen in dry ice/hexane or acetone.

Histological analysis was conducted as previously described (Tsukahara et al. 2002). In brief, frozen tissues were fixed in half strength Bouin's solution overnight and were embedded in paraffin. Cross‐sections that were 3‐*μ*m thick were stained with periodic acid‐Schiff counterstained with hematoxylin (PAS), alcian blue at pH 2.5 or pH 1.0, counterstained with Kernechtrot for neutral mucin, sialomucin, and sulfomucin staining, respectively. The cecal contents were diluted fivefold with saline and centrifuged at 10,000*g*. The IgA concentration in the supernatants was determined using a Rat IgA ELISA Quantitation kit (Bethyl Laboratories, Montgomery, TX), according to the manufacturer's protocols.

#### Animal experiment 3

The effects of GCL2505 on constipation that had been induced by loperamide in rats were investigated (Fig. S1C). The rats were allowed free access to drinking water and commercial nonpurified diet (MF, Oriental Yeast Co., Tokyo, Japan). After adaptation for 7 days, the rats were divided into three groups. Six rats each went into the following groups: (1) saline‐administered group (NTC group), (2) saline‐ and loperamide‐administered group, (control group), and (3) GCL2505‐ and loperamide‐administered group (GCL2505 group). The rats in the GCL2505 group were orally administered GCL2505 (10^9^ cfu/day, suspended in 0.3‐mL saline) throughout the experiment. NTC and control group rats were administered oral saline. Ten days after GCL2505 or saline administration, loperamide (Wako Chemical, Osaka, Japan) or saline was administered. Loperamide (1.5 mg/kg) suspended in distilled water was orally administered to the rats twice a day at 9:00 and 18:00 h on experimental day 1___5, while the rats in the NTC group were only administered distilled water in the same manner as those of the control and GCL2505 groups. Body weight and food intake were measured, and excreted feces were individually collected during the experimental period. The feces were thoroughly lyophilized, and the dry weight was measured. Cecal contents were collected on experimental day 6. Organic acid concentrations in the cecal contents were determined by the YMC‐Pack FA kit (YMC Co., Tokyo, Japan), according to the manufacturer's protocol.

### Tolerance to free bile acids

Bacteria were anaerobically cultured for 24 h in media containing 1% glucose, 2% glucose, 1% Bacto‐tryptone (BD, Franklin Lakes, NJ), 2% Bacto yeast extract (BD), 0.2% Na_2_HPO_4_, and 0.4% NaH_2_PO_4_. Bacterial cultures were inoculated (100 *μ*L) into 5 mL of the same broth that contained various concentrations of CA or DCA (Sigma Chemicals, St. Louis, MO). After 24 h, bacterial growth was monitored by measuring the absorbance of the culture broth at 660 nm.

### Statistics

Differences between the groups were determined using Student's *t*‐test or the Tukey–Kramer multiple comparison test. *P‐*values of <0.05 were considered statistically significant. Statistical tests were performed using Statcel2 software (Statcel2, OMS, Tokyo, Japan) or open‐source software R, Version 3.1.1 (http://cran.r-project/org). Data were expressed as mean ± standard error.

## Results

### Dynamics of GCL2505 and JCM 1217^T^ in the rat intestine (animal experiment 1)

**Figure 1:**
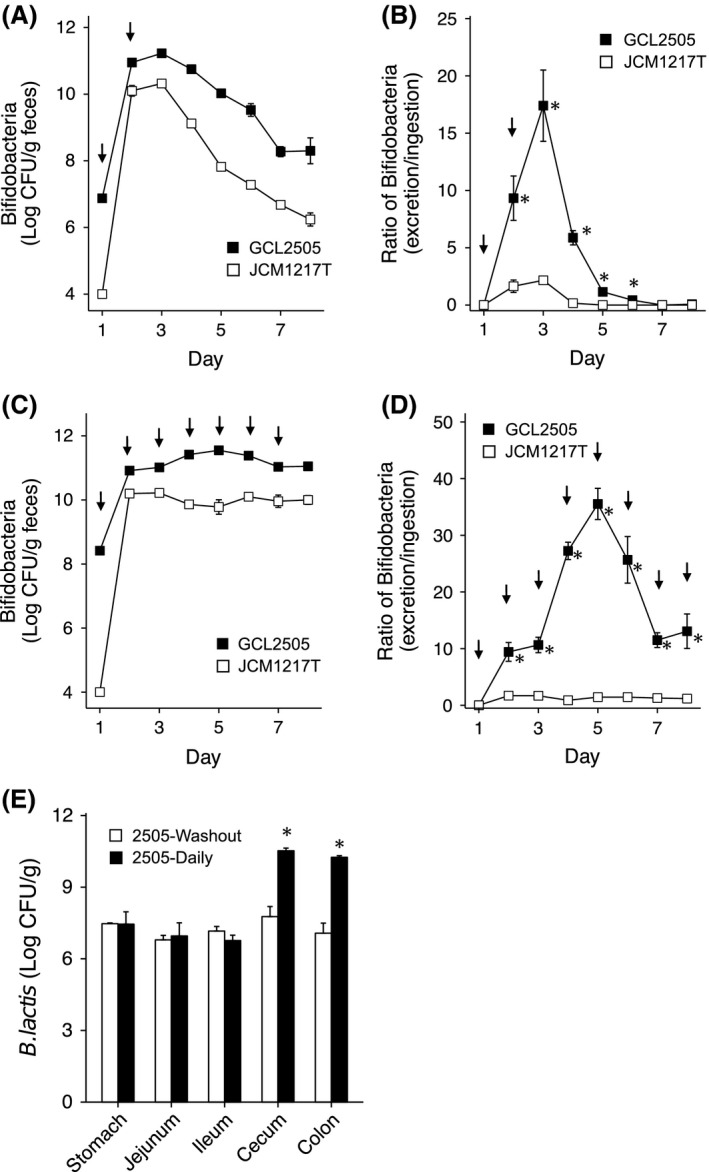
Dynamics of GCL2505 and JCM 1217T in the rat intestine. (A) Number of bifidobacteria in the feces excreted for 24 h from the transient administration groups. White squares represent the number *B. longum* in the JCM 1217T group (*N* = 5). Black squares represent the number of *B. animalis* ssp. *lactis* in the GCL2505 group (*N* = 4). (B) Proliferative ratio calculated from excreted and ingested bifidobacteria in the transient administration groups. (C) Number of bifidobacteria in the feces excreted for 24 h in the serial administration groups. White squares represent the number of *B. longum* in the JCM 1217T group (*N* = 5). Black squares represent the number of *B. animalis* ssp. *lactis* in the GCL2505 group (*N* = 6). (D) Proliferative ratio calculated from excreted and ingested bifidobacteria in the consecutive administration groups. (E) Number of *B. animalis* ssp. *lactis* in various intestinal contents in the GCL2505‐ingested rats (GCL2505‐transient group: *N* = 4, GCL2505‐consecutive group: *N* = 6). Arrows indicate bifidobacterial administration. Values are expressed as mean ± SE, *P < 0.05 (Student's t‐test).

The intestinal dynamics of *B. animalis* ssp. *lactis* GCL2505 and *B. longum* ssp*. longum* JCM 1217^T^ was investigated by species‐specific real‐time PCR on all fecal samples collected throughout the experiments. In the transient administration experiment (transient feeding groups), the levels of *B. animalis* ssp. *lactis* in feces reached 11.0 ± 0.08 log cfu/g feces on day 3 in the GCL2505‐transient feeding group, whereas the levels of *B. longum* in feces were significantly lower (10.3 ± 0.02 log cfu/g) in the JCM 1217^T^‐transient feeding group (Fig. 1A). In the consecutive administration experiment, the fecal levels of *B. animalis* ssp. *lactis* in the GCL2505‐consecutive feeding group were over 11 log cfu/g feces after administration and were maintained throughout the experiment, although those of *B. longum* in the JCM 1217^T^‐consecutive feeding group were significantly lower (approximately 10 log cfu/g feces, Fig. 1C). Figure 1B and D show the proliferation ratio of ingested bifidobacteria, which was calculated from the number of excreted and administered bifidobacteria. In both transient and consecutive administration experiments, GCL2505 showed over a 10‐fold proliferation. In contrast, JCM 1217^T^ showed a slight proliferation (approximately 2 fold).Figure 1E shows the level of *B. animalis* ssp. *lactis* in the intestinal tracts of the rats that were administered GCL2505. The number of *B. animalis* ssp. *lactis* in the transient feeding group was approximately 7 log cfu/g content, which indicated that the rats used in this experiment harbored endogenous *B. lactis*. In the stomach, jejunum, and ileum, there were no significant differences in the levels of *B. animalis* ssp. *lactis* between the transient and consecutive feeding groups. In the cecum and colon, the levels of *B. animalis* ssp. *lactis* in the consecutive feeding group were significantly higher than those in the transient feeding group.

### Effect of GCL2505 and JCM 1217^T^on mucin and IgA secretion (animal experiment 2)

**Figure 2:**
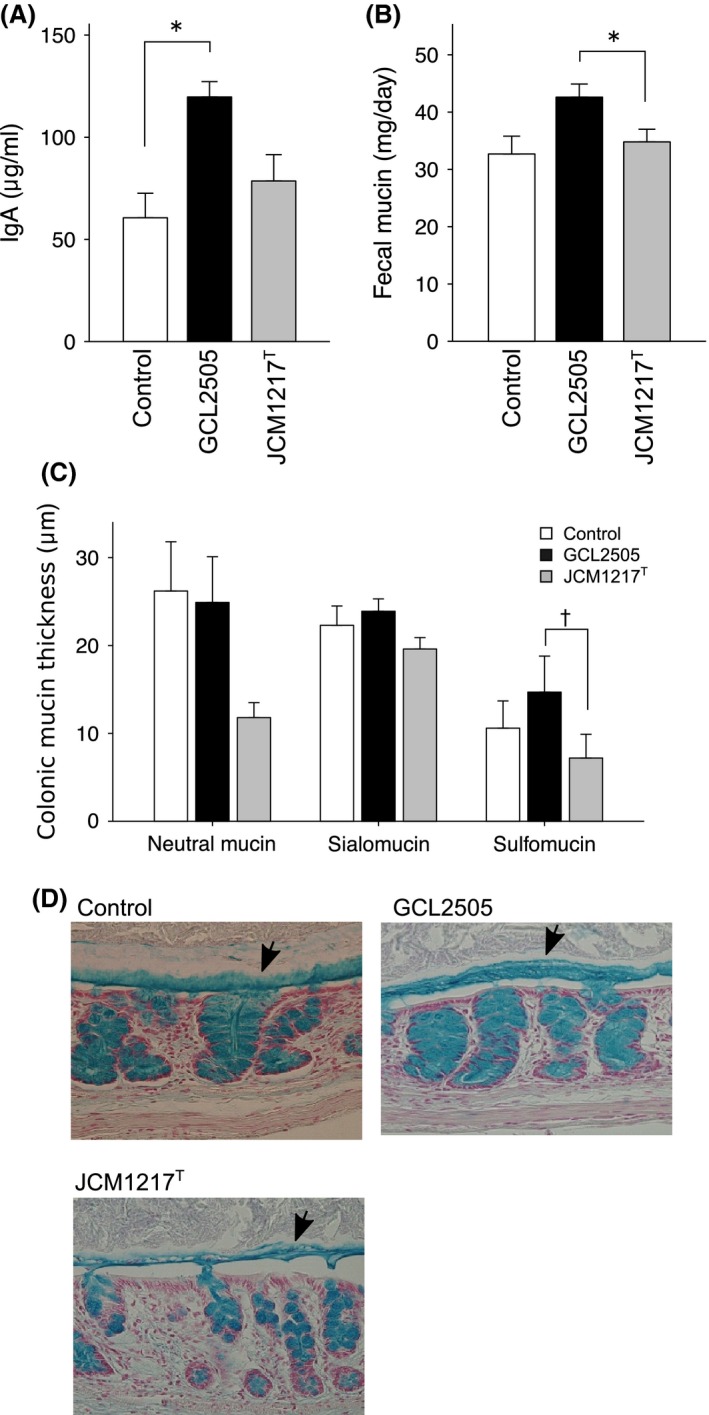
Effect of GCL2505 and JCM 1217Ton mucin and IgA secretion. (A) IgA concentration of cecal contents. (B) Fecal mucin excreted on day 7 of the ingestion period. (C) Colonic mucin thickness calculated from the histological section. (D) Representative photomicrographs of the distal colon stained by alcian blue (pH 1.0). Black arrows indicate the mucus layers of sulfomucin between the epithelium and digesta. Values are expressed as mean ± SE (*N* = 3–5), *P < 0.05, ^†^ *P* < 0.1 (Tukey–Kramer multiple comparison test).

Next, we examined variables concerning the intestinal physiology and environment. The cecal IgA level in the GCL2505‐administered group was significantly higher than that in the control group. In contrast, the cecal IgA level in the JCM 1217^T^‐administered group did not manifest any change compared with that in the control group (Fig. 2A). Mucin excretion on day 7 in the GCL2505‐administered group was significantly higher than that in the JCM 1217^T^‐administered group (Fig. 2B) and tended to be higher than that in the control (*P *>* *0.05). Histological analysis was performed to examine the colonic mucin thickness (Fig. 2C and D). Sulfomucin thickness of the colon in the GCL2505‐administered group tended to be higher than that in the JCM 1217^T^‐administered group. There was no difference in the mucin thickness of the small intestine and the number of goblet cells per crypt among the groups (data not shown).

### Laxative Effect of GCL2505 (Animal experiment 3)

**Figure 3:**
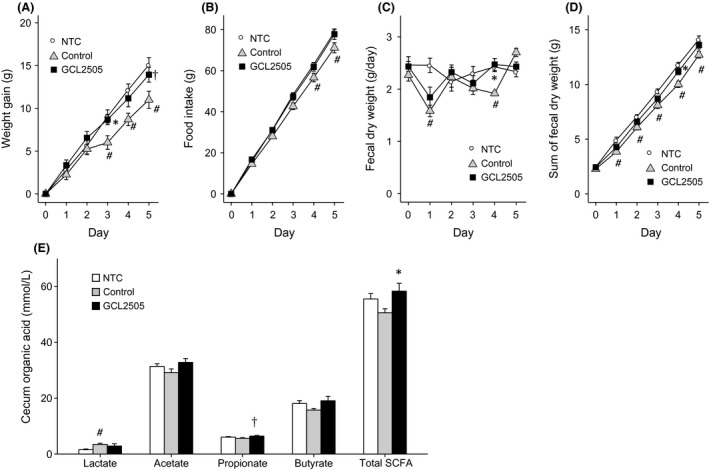
Laxative Effect of GCL2505. (A) Weight gain after loperamide treatment. (B) Sum of food intake after loperamide treatment. (C) Daily fecal excretion. (D) Sum of fecal excretion after loperamide treatment. (E) Organic acid concentration of cecal contents. Total SCFAs represents the sum of acetate, propionate, and butyrate. Values are expressed as mean ± SE (*N* = 6) *P < 0.05 compared with the control group, ^#^ *P* < 0.05 compared with the NTC group, ^†^ *P* < 0.1 compared with the control group (Tukey–Kramer multiple comparison test).

The laxative effect of GCL2505 was examined using a loperamide‐induced constipation model. Loperamide treatment suppressed weight gain and fecal excretion, although weight gain and fecal excretion were significantly elevated in the GCL2505‐administered group compared with that in the loperamide‐treated control group (Fig. 3A, C, and D). Loperamide treatment also suppressed food intake. In contrast, there was no significant difference in the food intake between GCL2505‐administered and saline‐administered (NTC) groups (Fig. 3B). SCFA concentration in the GCL2505‐administered group was significantly higher than that in the loperamide‐treated control group (Fig. 3E).

### Bile acid tolerance of GCL2505

**Figure 4:**
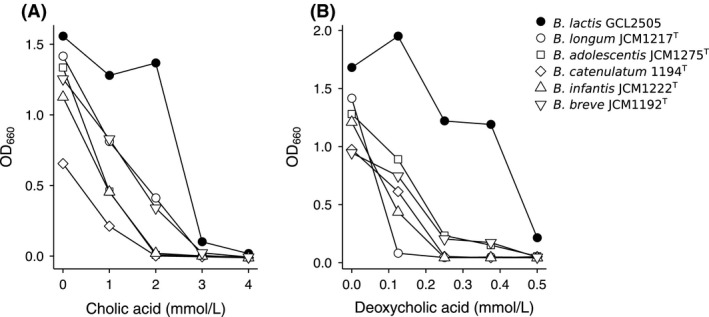
Bile acid tolerance of GCL2505. Growth of various bifidobacteria in the presence of different free bile acid concentrations was measured as OD660 after 24‐h culture. All data are representative of at least two independent experiments. (A) Cholic acid. (B) Deoxycholic acid.

To estimate the possible mechanism(s) involved in the growth of GCL2505 in the large intestine, free bile acid tolerance was investigated. GCL2505 showed a higher tolerance to cholic acid (CA), a predominant free bile acid in the human large intestine, than other bifidobacteria (Fig. 4A). The growth of GC2505 was unaffected in the presence of 0.25 mmol/L deoxycholic acid (DCA), which remarkably suppressed the growth of other bifidobacteria (Fig. 4B). In the presence of 0.5 mmol/L DCA, only GCL2505 manifested the proliferation.

## Discussion

GCL2505 showed significantly higher proliferation *in situ* than JCM 1217^T^ (Fig. 1 A–D). It has been shown that several strains of *B. animalis* ssp. *lactis* proliferates in the human intestine following ingestion, although the intestinal portion where *B. animalis* ssp. *lactis* grew was not precisely indicated in previous studies (Matsumoto 2000; Ishizuka et al. 2012; Watanabe and Isono 2012). Our study indicated that this remarkable proliferation of *B. animalis* ssp. *lactis* mainly occurred in the cecum (Fig. 1E). In the cecum of rats, where active fermentation occurs, GCL2505 actively participates in fermentation to proliferate in the cecum. The cecum of rats is believed to be equivalent to that of human ascending and proximal parts of the transverse colon in terms of sites for fermentation. The proliferation of *B. animalis* ssp. *lactis* in humans, therefore, occurs in the ascending and transversal colons. Several mechanisms underlie such an active proliferation of *B. animalis* ssp. *lactis* (namely GCL 2505) in the colon. One of the possibilities is the wide spectrum for carbohydrate utilization of *B. animalis* ssp. *lactis* because bifidobacteria are widely known to degrade various indigestible polysaccharides (Van den Broek et al. 2008). In this study, however, GCL2505 was shown to proliferate in rats fed AIN‐93G, which only contained corn starch and cellulose as carbohydrate sources (Reeves et al. 1993); nevertheless, it is well known that *B. animalis* ssp. *lactis* does not grow on starch, dextrin, or cellobiose as sole carbon sources (Scardovi et al. 1971). Comparative genomics has indicated that *B. animalis* ssp. *lactis* has fewer sugar transport systems and predicted genes for carbohydrate and polyol utilization than other bifidobacteria (Lee and O'Sullivan 2010). Therefore, carbon sources in the large intestine seem to have little direct effect on GCL2505 proliferation in the large intestine, and mechanisms other than sugar utilization should be involved in the proliferation of *B. animalis* ssp. *lactis* in the large intestine. Other possible mechanisms may be concerned with the sensitivity of *B. animalis* ssp. *lactis* to endogenous and exogenous growth inhibitors. Secondary bile acids in the large intestine are such compounds, although no report on the inhibitory effect of secondary bile acids on *B. animalis* ssp. *lactis* is currently available. Conjugated cholic acids, once released into the small intestine, are hydrolyzed into hydrophobic‐free cholic acids. Some bile acids escape from reabsorption at the terminal ileum, and they are converted into secondary bile acids that are highly hydrophobic and toxic to microorganisms in the large intestine (Ridlon et al. 2006). In this study, we demonstrated that GCL2505 showed higher resistance to free cholic acids, especially to DCA (Fig. 4). These results imply that resistance of GCL2505 to free cholic acids, particularly secondary bile acids, which are potent inhibitory agents in the large intestine for bacterial growth, can be attributable to its higher proliferation rate in the cecum. We propose that tolerance to free bile acids, especially to secondary bile acids such as DCA, should be considered when screening probiotic strains in addition to tolerance to gastric and conjugated bile acids and oxygen in future studies.

GCL2505 administration manifested a significant effect on IgA and mucin secretion in the gut. In contrast to GCL2505, such effects of JCM 1217^T^ were negligible. Several reports have shown that SCFAs are involved in the secretion of mucin and IgA in the colon (Shimotoyodome et al. 2000a; Hosono et al. 2003). Thus, the induction of mucin and IgA excretion by GCL2505 treatment can be explained, at least in part, by the elevation of luminal SCFAs, to which the proliferation of GCL2505 in the gut contributed. It is well established that the effects of probiotics on gut physiology are most likely strain‐ or at least species specific (Luyer et al. 2005; Kekkonen et al. 2008; Fukuda et al. 2011). The characteristics of probiotics responsible for strain‐ or species‐specific effects are still under investigation and largely unknown which. However, as discussed above, the level of proliferation in the large intestine may be related to the magnitude of this effect.

GCL2505 induced the thickening of the sulfomucin layer in the mucus layer of the proximal colon (Fig. 2C and D), but had no effect on the goblet cell density (number per crypt) and the thickness of the mucosal layer of the other intestinal portions (data not shown). It has been reported that butyrate plays a key role in sulfomucin synthesis (Tobisawa et al. 2010). However, no changes in butyrate concentration have been observed after GCL2505 treatment (Fig. 3E). Therefore, the induction of thickened sulfomucin by GCL2505 ingestion is obscure.

We evaluated the GCL 2505‐induced enhancement of mucin secretion in terms of preventing constipation. We used the loperamide‐induced constipation model because loperamide treatment decreases mucus thickness of the colon (Shimotoyodome et al. 2000b). GCL2505 administration significantly elevated fecal excretion (total mass of the feces and frequency of defecation) compared with fecal excretion in the loperamide‐treated control group (Fig. 3C and D). Increased mucin production and excretion apparently prevent animals from constipation induced by loperamide. SCFAs also play significant roles in bowel movement and defecation. It has been well characterized that SCFAs stimulate colonic motility via 5‐HT and peptide YY release (Cherbut et al. 1998; Fukumoto et al. 2003). In addition, a recent report has shown that propionate induces epithelial ion secretion by a non‐neuronal acetylcholine response (Yajima et al. 2011). In this study, GCL2505 administration elevated the total SCFA and propionate concentration in the cecum (Fig. 3E), probably resulting in the amelioration of loperamide‐induced constipation as well as the enhancement of mucin secretion.

## Conclusions

The purpose was to investigate the intestinal dynamics of *B. animalis* ssp. *lactis* GCL2505 and *B. longum* JCM 1217^T^ in addition to the physiological response of the host. The main findings are summarized as follows: (1) GCL2505 actively proliferates in the large intestine; (2) the level of proliferation in the intestine affects the physiological response of the host such as IgA production and mucin secretion, which possibly affect gastrointestinal functions such as bowel movements to prevent constipation; and (3) GCL2505 exhibits high tolerance to secondary bile acids, which explains, at least in part, its higher rate of proliferation in the large intestine.

## Conflict of Interests

The authors declare that they have no competing interests.

## Supporting information


**Figure S1.** The design of animal experiments. (A) The design of animal experiment 1. (B) The design of animal experiment 2. (C) The design of animal experiment 3.
