# Supplementary material for: Effect of Bifidobacterium animalis subsp. lactis GCL2505 on the physiological function of intestine in a rat model
Source: Food Sci Nutr. 2016 Feb 8;4(6):782–90. doi: 10.1002/fsn3.344 (PMC5090641; doi:10.1002/fsn3.344)
Supplement: Supplementary file 1 — Figure S1. The design of animal experiments. (A) The design of animal experiment 1. (B) The design of animal experiment 2. (C) The design of animal experiment 3. [file FSN3-4-782-s001.pdf]

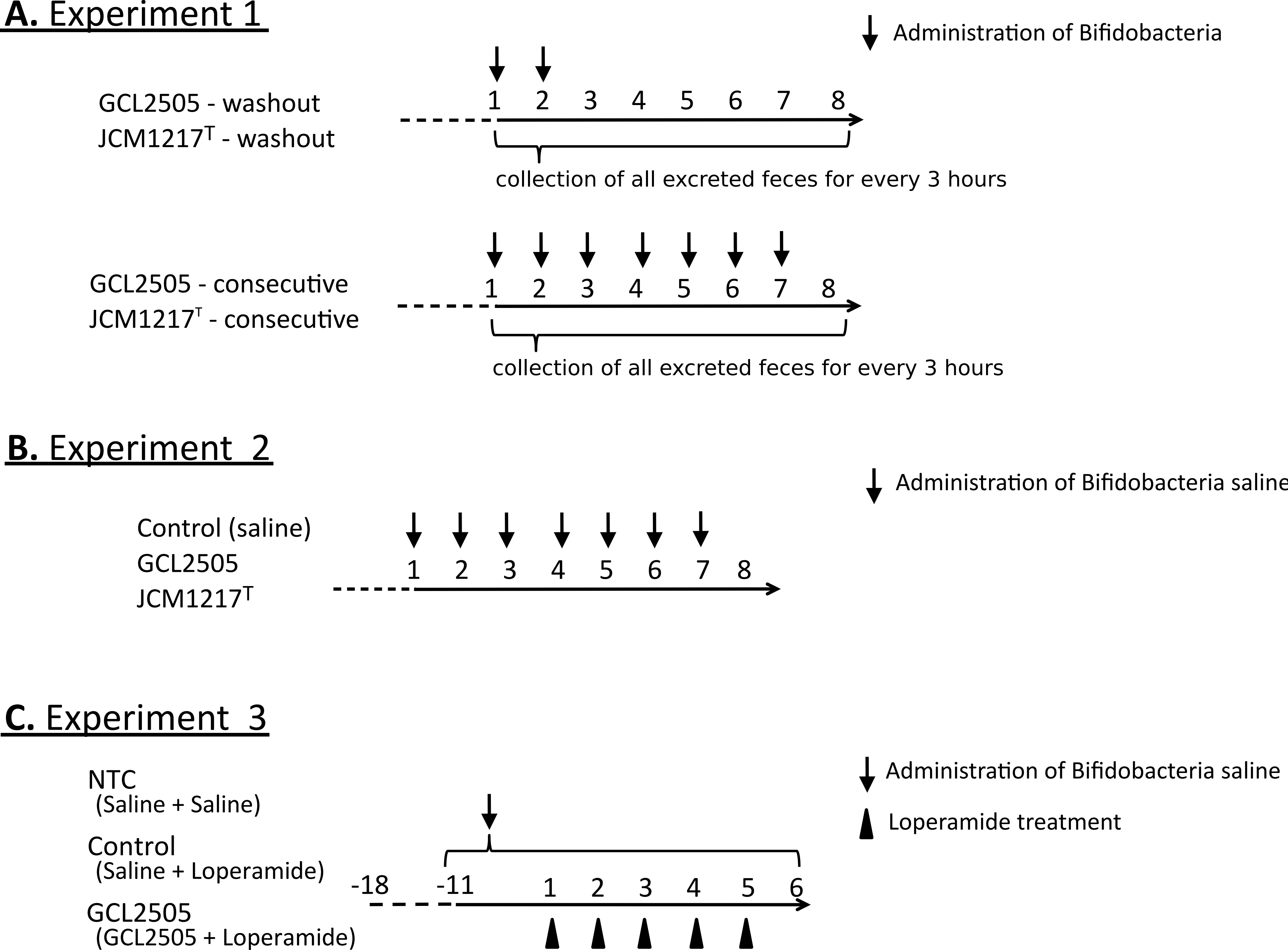

A. Experiment 1
Administration of Bifidobacteria
GCL2505 - washout
JCM1217^T - washout
1 2 3 4 5 6 7 8
collection of all excreted feces for every 3 hours
GCL2505 - consecutive
JCM1217^T - consecutive
1 2 3 4 5 6 7 8
collection of all excreted feces for every 3 hours
B. Experiment 2
Administration of Bifidobacteria saline
Control (saline)
GCL2505
JCM1217^T
1 2 3 4 5 6 7 8
C. Experiment 3
Administration of Bifidobacteria saline
Loperamide treatment
NTC
(Saline + Saline)
Control
(Saline + Loperamide)
GCL2505
(GCL2505 + Loperamide)
-18 -11 1 2 3 4 5 6
